# *cspA* Influences Biofilm Formation and Drug Resistance in Pathogenic Fungus *Aspergillus fumigatus*

**DOI:** 10.1155/2015/960357

**Published:** 2015-03-03

**Authors:** Zhongqi Fan, Zhe Li, Zongge Xu, Hongyan Li, Lixiang Li, Cong Ning, Lin Ma, Xiangli Xie, Guangyi Wang, Huimei Yu

**Affiliations:** ^1^Department of Hepatobiliary Pancreas Surgery, The First Hospital, Jilin University, Changchun 130021, China; ^2^Department of Pathology and Pathophysiology, School of Basic Medical Sciences, Jilin University, Changchun 130021, China; ^3^Department of Experimental Center of Pathogenobiology Immunology, School of Basic Medical Sciences, Jilin University, Changchun 130021, China; ^4^College of Plant Sciences, Jilin University, Changchun, Jilin 130062, China

## Abstract

The microbial cell wall plays a crucial role in biofilm formation and drug resistance. *cspA* encodes a repeat-rich glycophosphatidylinositol-anchored cell wall protein in the pathogenic fungus *Aspergillus fumigatus*. To determine whether *cspA* has a significant impact on biofilm development and sensitivity to antifungal drugs in *A. fumigatus*, a Δ*cspA* mutant was constructed by targeted gene disruption, and we then reconstituted the mutant to wild type by homologous recombination of a functional *cspA* gene. Deletion of *cspA* resulted in a rougher conidial surface, reduced biofilm formation, decreased resistance to antifungal agents, and increased internalization by A549 human lung epithelial cells, suggesting that *cspA* not only participates in maintaining the integrity of the cell wall, but also affects biofilm establishment, drug response, and invasiveness of *A. fumigatus*.

## 1. Introduction

*Aspergillus fumigatus* is a major cause of infection in individuals with a compromised immune system, including patients undergoing treatment for leukemia, HIV, or organ transplant, and in those suffering from an underlying disease such as cystic fibrosis [1, 2]. Aspergilloma and invasive aspergillosis are the main forms of* A. fumigatus* infection, both of which are characterized by high mortality rates (50–95%) and the germination of conidia and subsequent invasion of mycelia into tissues such as human pulmonary alveoli [3].

Under natural conditions, such as in human bodies, most fungal and bacterial pathogens are present as part of a biofilm, which contributes to their decreased response to antibiotics and host immune defenses compared with bacteria in the planktonic state [4]. Several* in vitro* studies have shown that* A. fumigatus* biofilms usually contain parallel-packed hyphae and that some cultures even contain self-produced extracellular matrix (ECM) [5–7]. Recently, two aspergilloma specimens were dissected [8] and observed to contain hyphae surrounded by ECM. This presentation is clinically recognized as the primary evidence of biofilm formation by* A. fumigatus*.

Despite ample evidence of biofilm formation by* A. fumigatus*, there is very little research on the mechanisms involved in this process. Among fungi,* Candida albicans* biofilm formation has been the most intensively studied system. Proteins localized on the cell wall, including Hwp1 and Als3, appear to be closely linked to biofilm formation in this species [9]. It is reasonable to suppose that cell wall proteins also participate in the formation of biofilm by* A. fumigatus*. Glycophosphatidylinositol- (GPI-) anchored proteins are the main cell wall proteins. The antifungal agent E1210 interrupts GPI biosynthesis and suppresses biofilm formation by* C. albicans* [10].

*cspA* encodes cell surface protein A, a 433-aa protein containing a putative leader sequence and a specific GPI modification site in* A. fumigatus*. A previous study also found that* cspA* lacked recognizable catalytic domains, and the only homologous gene regions were in* Aspergillus* species. Deletion of* cspA* resulted in reduced adhesion to ECM, along with an increase in exposed chitin on the cell wall in* A. fumigatus* [11]. Therefore, we hypothesized that the GPI-anchored protein* cspA* may influence biofilm formation through its effects on the cell wall.

In this study, we constructed a Δ*cspA A. fumigatus* strain by targeted gene disruption mediated by* Agrobacterium tumefaciens*, along with a complemented mutant strain generated by homologous introduction of an intact* cspA* sequence. Deletion of* cspA* changed colony and conidia morphology, reduced biofilm formation, decreased resistance to antifungal agents, and increased internalization by A549 human lung epithelial cells. These findings suggested that* cspA* not only participates in maintaining the integrity of the cell wall, but also plays an important role in biofilm establishment, drug resistance, and invasiveness of* A. fumigatus*.

## 2. Materials and Methods

### 2.1. Strains and Culture Conditions

*A. fumigatus* wild type (WT) strain Af293 was used in this study. The WT strain, the Δ*cspA* mutant, and the complementation strain (*cspA*C) were cultured in potato dextrose broth (PDB; BD Biosciences) medium and incubated at 37°C. Conidia were harvested from strains grown on potato dextrose agar (PDA) plates.

### 2.2. Construction of the Δ*cspA* and* cspA*C Strains of* A. fumigatus*

The plasmid used to generate the Δ*cspA* mutant strain was constructed using the* A. fumigatus cspA* sequence (locus tag AFUA 3G08990 in GenBank accession NC_007196.1). Based on this sequence, we designed primers to amplify the* cspA* LB and RB regions. The primers for* cspA* LB were 5′-GCG-GTA-TTG-TTG-TAA-GGT-CG-3′ and 5′-GTG-GAG-TCG-CTT-GAT-GTT-T-3′. The primers for* cspA* RB were 5′-GCT-GGT-ATC-TGG-GTT-GTC-AT-3′ and 5′-ACT-TTG-AGC-GTC-TCC-TCT-G-3′. To construct the* cspA* gene deletion plasmid, the* cspA* LB and RB regions were amplified from* A. fumigatus* genomic DNA. The* cspA* LB and* cspA* RB fragments were ligated into the upstream and downstream regions of the hygromycin B phosphotransferase resistance gene (*hph*) in the pXEH vector, respectively, generating plasmid pXEH-Δ*cspA*. An* A. fumigatus* Af 293 Δ*cspA* mutant strain was then generated using the pXEH-Δ*cspA* vector by* A. tumefaciens*-mediated transformation (ATMT), as described previously [12].

To ensure that the mutant phenotype obtained could be attributed to the desired deletion, the* cspA* deletion was complemented by integration of the Af 293* cspA* gene to generate a complementation strain,* cspA*C. Briefly, a DNA fragment containing the* cspA* promoter, open reading frame, and terminator was cloned and inserted into the pCB1532 vector containing the phleomycin resistance gene (*phl*), generating complementation vector pCB1532-*cspA*C. The* cspA*C strain was then generated by ATMT using the complementation vector.

### 2.3. Growth Rate and Morphological Observation

Strains were incubated at 37°C. Conidia were harvested from strains grown on potato dextrose agar (PDA) plates and resuspended in sterilized dH_2_O to a final concentration of 1 × 10^7^ spores/mL. To measure growth rates, 10^5^ conidia in 10 *μ*L dH_2_O were spotted onto the center of a plate of PDA and incubated at 37°C for 4 days; then the changes of the color, shape, and diameter of colonies were observed at various time points. In order to compare the microscopic morphology among the stains, they were inoculated on the PDA agar block, after the emergence of the pending hyphae and conidia (about 30 h at 37°C), whose morphology was observed under microscope at 400x magnification.

### 2.4. Crystal Violet Assay

The 96-well plates were inoculated with 100 *μ*L of PDB containing 10^5^, 10^4^, and 10^3^ per well and incubated for 20 h at 37°C, and at least 5 replicates of each parameter were adapted. The residual medium was abandoned carefully, and biofilms were washed 3 times with PBS to remove mycelia or conidia floating in the medium. After the extra PBS was aspirated, the biofilms were fixed in formaldehyde for 15 min and washed 3 times with PBS. Biofilms were stained by 0.5% (w/v, methanol as solution) crystal violet for 30 min at room temperature. The solution was discarded. The microplates were soaked in dH_2_O until the floating color faded. After the rinsing 3 times, 96-well plates dried naturally. Then 100 *μ*L of 95% (v/v) ethanol was added into each well and slightly shaken for 10 min until the crystal violet dissolved uniformly. The biofilm quantification was performed by Microplate Reader (Molecular Devices), and absorbance at a wavelength of 570 nm (*A*_570_) was reported.

### 2.5. Biofilm Formation and Fluorescence Microscopy

To generate an* A. fumigatus* biofilm, 200 *μ*L of each* A. fumigatus* strain culture grown in PDB was inoculated onto sterilized 1 cm^2^ coverslips arranged in 24-well plates (Wuxi NEST Biotechnology Co., Ltd.). The concentration of* A. fumigatus* conidia was 10^5^/mL. Following incubation for 16 h at 37°C, the supernatant was carefully removed and the biofilms were washed three times with PBS. Each well was stained with 150 *μ*L of 20 *μ*M probe FUN1 (Life Technologies), which stains the cytoplasm with a diffusely distributed green fluorescence, for 30 min at room temperature in the dark. Biofilms were washed three times with PBS and then observed using a fluorescence microscope (Olympus, IX71) at 100x magnification.

### 2.6. Scanning Electron Microscopy (SEM)

Biofilm samples of the WT, Δ*cspA*, and* cspA*C strains were prepared as for fluorescence microscopy. The coverslips were then prefixed with 2% (wt/vol) glutaraldehyde at 4°C for 10 h and then metalized with gold and observed by scanning electron microscopy.

### 2.7. Quantitative Real-Time PCR (qRT-PCR)

A total of 10^8^ conidia of the WT, Δ*cspA*, and* cspA*C strains were incubated in 10 mL of PDB in a rotary shaker at 37°C for 24 h. The mycelia were collected and total RNA was extracted according to the Trizol (Invitrogen) method. cDNA was generated using a Transgen reverse transcription kit according to the manufacturer's instructions. Levels of* cspA* mRNA transcript were then investigated in each of the strains by real-time PCR using the fluorescent reporter SYBR Green (ABI, 4385612) and an ABI 7300 thermocycler (Applied Biosystems). The* gapdh* gene was used as an internal control. The primers for* cspA* were 5′-ATG-ATG-CTC-CAC-CTG-ACC-T-3′, 5′-AAG-TCG-GAA-CCA-GAG-GAT-3′. The primers for GAPDH were 5′-ATT-CCT-TCT-CTC-AAC-GGC-3′, 5′-ACA-ACA-TCG-TCC-TCA-GTG-3′.

### 2.8. Internalization Assay

Human A549 lung epithelial cells were incubated in microwell plates and co-incubated for 48 h at 37°C under 5% CO_2_ (approximately 8000 cells per well) supplemented with 10% foetal bovine serum (FBS, Hyclone) and RMPI 1640 (GIBCO). Then the medium was aspirated, followed by thorough rinsing three times using sterile PBS. Subsequently, 1 × 10^5^ conidia resuspended were cocultured with A549 to induce internalization for 4 h under the same cultured conditions. Each experimental parameter was repeated three times. Afterwards, the cell monolayers were washed three times with PBS. 1640 plus 10% FBS containing 20 *μ*g/mL nystatin was used to dispose each well for 3.5 h to kill noninternalized conidia. Then the cell monolayers were treated with 100 *μ*L liquid comprising PBS and 0.1% Triton X-100 for 15 min at 37°C to induce cell lysis and the release of internalized conidia. The released conidia were diluted and incubated on PDA at 37°C for 24~36 h. The internalization rate was determined to be the percentage of monocolonies compared to the initial inoculum of conidia.

### 2.9. Antifungal Agents and End-Point Susceptibility Testing

Planktonic cell minimum inhibitory concentrations (PMICs) were examined according to the National Committee for Clinical Laboratory Standards M38-A standard methodology (NCCLS, 2002). The antifungal agents itraconazole (ICZ), amphotericin B (AmB), and 5-fluorocytosine (5-FC) were prepared as stock solutions and diluted in PDB to working concentrations. ICZ and AmB were serially diluted twofold to produce a concentration range of 0.0156–16 *μ*g/mL, while the concentration of 5-FC was 0.0625–64 *μ*g/mL. The pathogen was challenged for 24 h at 37°C. Following incubation, all wells were treated with 100 *μ*L of PBS containing 1 *μ*M 2,3-bis-(2-methoxy-4-nitro-5-sulfophenyl)-2H-tetrazolium-5-carboxanilide and 1 *μ*M menadione for 3 h. Absorbance was examined at a wavelength of 490 nm (*A*_490_). Sessile cell minimum inhibitory concentrations (SMICs) were determined as 50% and 90% reduction in *A*_490_ compared with the untreated control.

### 2.10. Statistical Analysis

All experiments were repeated at least three times. The means ± standard deviations of the colony diameters, crystal violet density, and the internalized rate were determined using GraphPad Prism 5 software. Data were analyzed using the repeated measures of SPSS 16.0. *P* < 0.05 was considered statistically significant.

## 3. Results

### 3.1. Disruption and Complementation of the* A. fumigatus cspA* Gene

**Figure 1:**
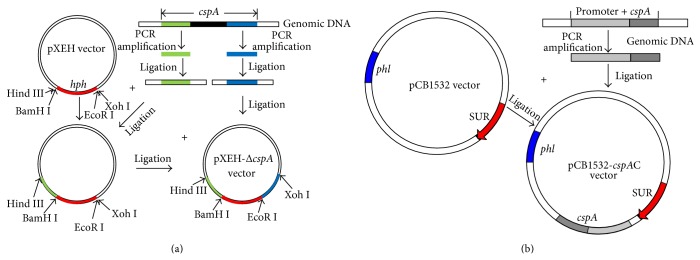
The constructed schematic of Δ*cspA* and* cspA*C in* A. fumigatus* mutant. (a) The constructed schematic of Δ*cspA* vector. The* cspA* gene was replaced by hph in the pXEH vector. (b) The constructed schematic of* cspA*C vector. The fragment containing the* cspA* promoter and ORF was cloned and inserted into the pCB1532 vector with phl.

**Figure 2:**
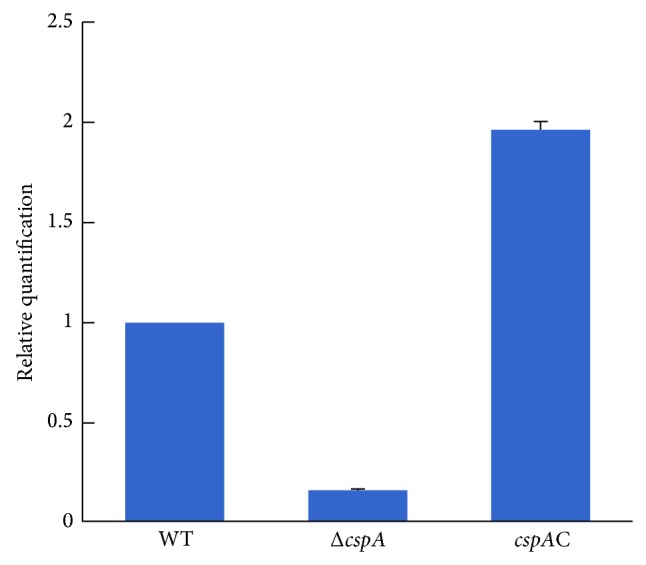
*cspA* gene disruption changes the morphology of* A. fumigatus*. A total of 10^8^ conidia were incubated in PDB in a rotatory shaker for 24 h. Total RNA was extracted based on Trizol method, and it was reversed to cDNA. The transcription level of* cspA* was evaluated by real-time PCR.

The construction of the plasmid for generation of the Δ*cspA* strain is outlined in Figure 1(a), while construction of the complementation plasmid is outlined in Figure 1(b). At 4 days after transformation of the respective plasmids by ATMT, we observed colonies on the selection plates. The downregulated expression of* cspA* in the mutant strain and restored expression in the complementation strain were confirmed by real-time PCR. Compared with the WT, there was a 4.7-fold decrease in transcription of* cspA* in the Δ*cspA* strain, while transcription increased 2-fold in the* cspA*C strain (Figure 2).

### 3.2. Disruption of* cspA* Changes Colony and Conidial Morphology of* A. fumigatus*

**Figure 3:**
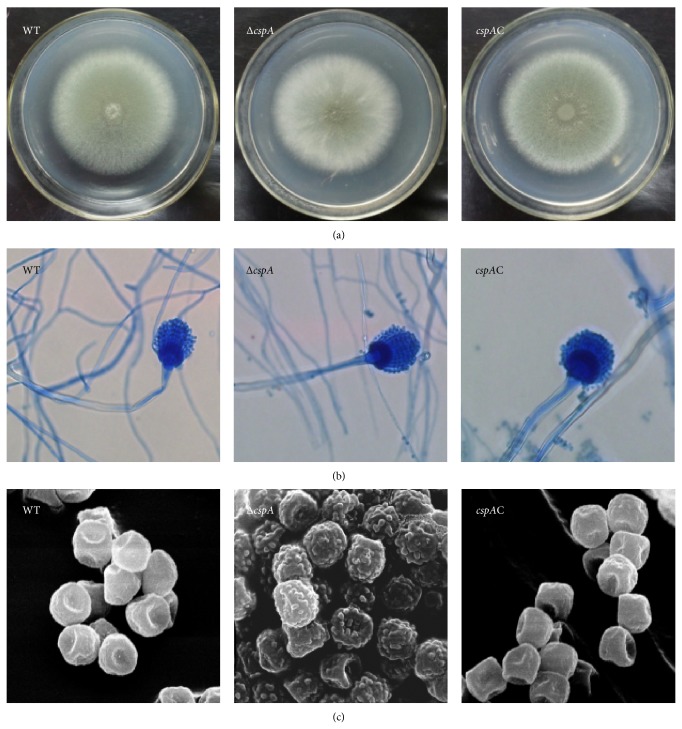
*cspA* gene disruption changes the morphology of* A*.* fumigatus*. (a) Conidia of* A. fumigatus* 293T wild type (WT), Δ*cspA*, and* cspA*C were inoculated and cultured in 37°C. 10^5^ conidia in 10 *μ*L were spotted in the center of PDA plates and cultured for 4 d. The colonial morphology was observed when cultured for 72 h. (b) The coverslips were also stained by lactophenol cotton-blue and observed by light microscope immediately. The data exhibited here are the mean ± SE (*n* = 3 for every experiment). The statistical differences existing between mutants and WT are analyzed with *t*-test. ^*^ *P* < 0.05. (c) The coverslips covered on PDA cubes with mycelia were transferred and prefixed with 2% glutaraldehyde at 4°C for 10 h and then metalized with gold and investigated by scanning electron microscope.

Differences in morphology between the WT, Δ*cspA* mutant, and* cspA*C strains were examined by inoculating conidia from each strain onto PDA and observing their morphology by light microscopy and SEM. There was a significant color difference between the WT and Δ*cspA* mutant, with the mutant strain showing reduced pigmentation compared with the WT strain (Figure 3(a)). The* cspA*C strain was similar in appearance to the WT (Figure 3(a)). However, there was no obvious difference in morphology of the conidia and hyphae of the three strains under light microscopy (Figure 3(b)). Under SEM, the conidia of the Δ*cspA* strain had a rougher surface with more ornaments compared with the smooth appearance of the WT and* cspA*C strains (Figure 3(c)).

### 3.3. Disruption of* cspA* Slightly Inhibits Growth of* A. fumigatus*

**Figure 4:**
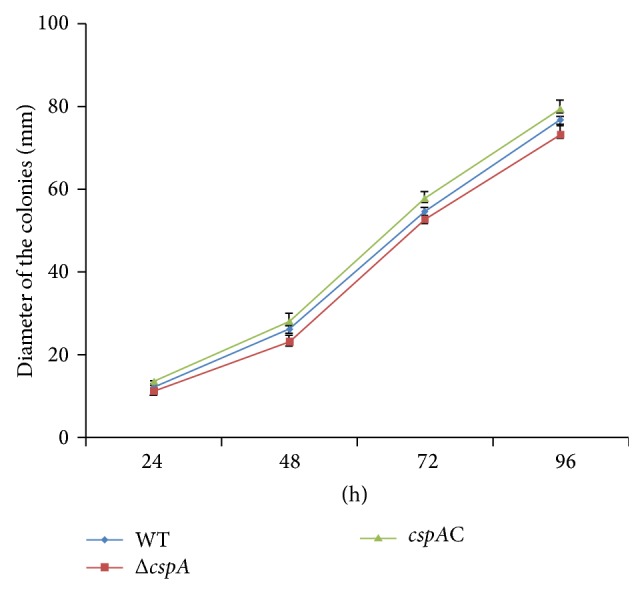
*cspA* gene disruption changes the growth of* A. fumigatus*. Conidia of* A. fumigatus* 293T (WT), Δ*cspA*, and* cspA*C were inoculated and cultured in 37°C. 10^5^ conidia in 10 *μ*L were spotted in the center of PDA plates and cultured for 4 d. The colonial diameter was measured each day, and the data was used to prepare a growth rate curve.

To investigate whether* cspA* is associated with the growth of* A. fumigatus*, a known amount of conidia was spotted onto PDA, and colony diameters were measured daily. A growth curve was established based on the diameters. Over the first 72 h, no obvious differences in growth were observed for the three strains (Figure 4). However, the diameter of the Δ*cspA* colony (73.2 ± 2.06 mm) was significantly (*P* < 0.05) smaller than that of the WT (76.8 ± 0.74 mm) when measured at 96 h after inoculation. The diameter of* cspA*C was akin to WT, with a colony diameter of 79.4 ± 2.04 mm at 96 h after inoculation (*P* > 0.05) (Figure 4).

### 3.4. Disruption of* cspA* Contributes to Reduced Biofilm Formation

#### 3.4.1. Crystal Violet Assay

**Figure 5:**
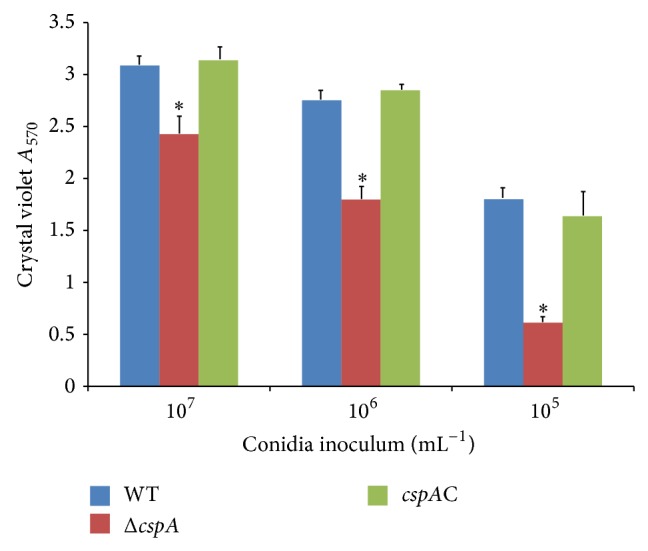
*cspA* gene disruption postpones* A. fumigatus* biofilm development. Each of 96-well plates was inoculated with 100 *μ*L of PDB containing different concentrations of conidia of WT or mutants per well and incubated for 20 h at 37°C. Then they were stained by 0.5% crystal violet for 30 min at room temperature, and absorbance at a wavelength of 570 nm (*A*_570_) was examined. The data exhibited here are the mean ± SE (*n* = 5-6 for every experiment). The statistical differences existing between mutants and WT are analyzed with *t*-test. ^*^ *P* < 0.05.

Crystal violet can bind to the components of cell walls and to the extracellular polysaccharide secreted during biofilm formation. At final spore concentrations of 10^6^, 10^5^, and 10^4^ spores/mL, the *A*_570_ values for the WT strain were 3.16 ± 0.20, 2.80 ± 0.18, and 1.81 ± 0.26, respectively. At the corresponding spore concentrations, the *A*_570_ readings for Δ*cspA* were 2.51 ± 0.47, 1.77 ± 0.20, and 0.64 ± 0.06, respectively. These observed decreases in the amount of bound crystal violet were significant (*P* < 0.05) (Figure 5). An increase in biofilm formation compared with the WT was observed for the complementation strain, with *A*_570_ values of 3.16 ± 0.26, 2.87 ± 0.28, and 1.61 ± 0.27 for the respective spore concentrations (Figure 5).

#### 3.4.2. Fluorescence Microscopy and SEM

**Figure 6:**
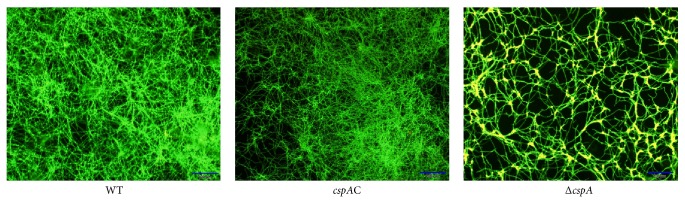
*cspA* gene disruption renders* A. fumigatus* different hyphal crosslinking. 200 *μ*L PDB containing 2 × 10^4^ conidia was inoculated per well of 24-well plate and cultured for 16 h at 37°C. Then each sample was stained by 20 *μ*M FUN1 probe. Fluorescence microscope was adopted.

**Figure 7:**
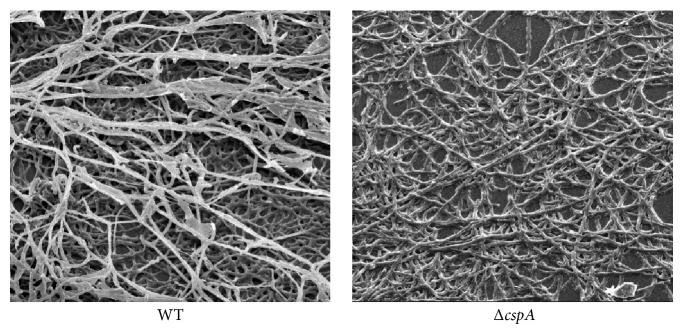
*cspA* gene disruption reduces* A. fumigatus* biofilm architecture. 2 × 10^4^ conidia were inoculated per well of 24-well plate containing 200 *μ*L PDB and cultured for 16 h at 37°C. Then samples were desiccated naturally and observed by SEM.

Probe FUN1 was used to observe the morphology of the filaments under a fluorescence microscope. Disruption of* cspA* contributed to the sparse, irregular arrangement of hyphae, which formed almost circle-like connections (Figure 6). Conversely, WT hyphae formed several clusters of parallel interwoven filaments forming an acute angle (Figure 6). When analyzed by SEM, a similar hyphal morphology was observed (Figure 7). Interestingly, nodules appeared at the intersections of overlapping hyphae in the Δ*cspA* mutant. The WT strain was rich in a membrane-like substance surrounding hyphae, which did not appear to be present in the Δ*cspA* strain (Figure 7).

### 3.5. Disruption of* cspA* Sensitizes* A. fumigatus* to Antifungal Agents

PMICs and SMICs were examined by end-point susceptibility testing. Of the three antifungal agents examined, the most significant differences in susceptibility of the three strains were observed in response to exposure to 5-FC. The PMICs for this agent for the WT, Δ*cspA*, and* cspA*C strains were 0.5 *μ*g/mL, <0.125 *μ*g/mL, and 0.25 *μ*g/mL, respectively. However, no differences were observed between the strains when treated with AmB or ICZ. In contrast to the planktonic cells, sessile hyphae were much more sensitive to AmB and ICZ. Using AmB, SMIC_50_ results were 32, 16, and 32 *μ*g/mL for the wild type, Δ*cspA*, and Δ*cspA*C strains, respectively, while the SMIC_90_ values were 64, 32, and 64 *μ*g/mL, respectively. Meanwhile, in response to ICZ, the SMIC_50_ results were 8, 1, and >32 *μ*g/mL for the WT, Δ*cspA*, and Δ*cspA*C strains, respectively, while the SMIC_90_ values were 16, 2, and >32 *μ*g/mL for the three strains, respectively. However, no difference was observed between the strains in response to 5-FC treatment (>256 *μ*g/mL) (Table 1).

### 3.6. Disruption of* cspA* Promotes* A. fumigatus* Internalization into A549 Cells

We used an A594 cell-conidia coculturing technique to examine the invasiveness of* A. fumigatus* and to observe its ability to escape from host immune cells. The internalization rate of the WT strain was 0.29 ± 0.02, and that of the Δ*cspA* mutant was 0.70 ± 0.09 (*P* < 0.05).

## 4. Discussion

*A. fumigatus* is an opportunistic fungus capable of surviving under various environmental conditions, which contributes to it being the primary cause of invasive aspergillosis in liver transplant recipients amongst the* Aspergillus* species [2].* CspA*, containing a GPI-associated site, is a cell wall protein found only in* Aspergillus* species and is present in multiple copies in the genome of* A. fumigatus*. Als proteins, also characterized as GPI-anchored proteins in* C. albicans*, play a critical role in biofilm formation. Thus, to unravel the relationship between* cspA* and biofilm formation in* A. fumigatus*, we constructed a Δ*cspA* mutant strain by targeted gene disruption, along with a complemented* cspA* strain (*cspA*C) by homologous introduction of a complementation plasmid. The Δ*cspA* mutant strain was examined by real-time PCR, and a 5-fold reduction in expression of* cspA* was identified. This finding confirmed the validity of this method for achieving targeted gene disruption of filamentous fungi, which is a prerequisite for studying fungal gene function.

Compared with the WT strain, the Δ*cspA* mutant showed a reduction in the grey-green pigmentation that is common to this species, with more ornaments on the conidial cell surface observed by SEM. The latter feature was consistent with a previous study, providing direct evidence of the function of* cspA* in the cell wall. However, almost no alteration of pigment was observed in the previous work [11]. This discrepancy is likely caused by differences in the strains used and the culture conditions. A recent study showed that two kinds of pigment are generated by* A. fumigatus*: pyomelanin, which binds to the cell wall of hyphae and imparts a brown coloration, and DHN melanin, which has an affinity for conidia, rendering them gray-greenish [13]. There are two major factors that might account for the observed decrease in pigment. The first is a decrease in the number of conidia; however, this can be excluded by the results of both culturing and SEM. The second reason is loss or poor production of melanin. The amount of melanin and the roughness of the conidial surface appear to be closely related [14–16]. Rather than being formed as a solid layer anchored to the cell wall, the melanin of fungi is produced as granules that are held in place by scaffolds constructed out of cell wall components, such as chitin [17]. In* Cryptococcus neoformans*, deletion of chitin synthase (Chs3) and its regulator (Csr2) contributed to a decrease in melanin retention [18]. Furthermore, disruption of chitin synthase resulted in the failure to produce melanin by* C. albicans* [19]. Together, these findings suggest that* cspA* might affect cellular melanin levels either by influencing melanin retention or moderating its synthesis. Thus, further research is necessary to elucidate the link between* cspA* and melanin in* A. fumigatus*.

Obvious differences in biofilm density existed between the WT, Δ*cspA* mutant, and* cspA*C complementation strains, confirming a role for* cspA* in* A. fumigatus* biofilm formation. Biofilms are involved in 65–80% of chronic clinical infections in humans and represent the sessile stage of most microorganisms. During biofilm formation, three landmark events should be taken into consideration: adherence to the surrounding substances, correct hyphal cross-linking, and sufficient ECM production [20, 21].

Emerging evidence suggests that certain cell wall proteins function as adhesins as well as effectors in biofilm formation. The disruption of RodA resulted in decreased adherence of* A. fumigatus* to epithelial cells and material, such as collagen and albumin, with higher levels of* rodA* transcription recorded in biofilms than in conidia. Meanwhile, GPI proteins play an important role in the adherence to epithelial and endothelial cell lines of* C. albicans* [9, 22]. Based on this evidence, we predicted that* cspA*, characterized as a cell wall GPI protein, was a potential adhesin. Our findings showed that the biofilm formed by the Δ*cspA* strain was more easily detached by washing than that of the WT strain and that the mutant took longer to attach to the bottom of the microwell plates (data not shown). Reduced adherence to the ECM by a Δ*cspA* mutant was also confirmed by another study [11].

Furthermore, we used Fun1 labeling of the hyphal wall to observe filament morphology. Disruption of* cspA* contributed to a sparse, irregular arrangement of hyphae, which formed almost circle-like connections instead of the classic bundles of parallel filaments intersecting and forming acute angles [5]. This abnormal hyphal arrangement might be the cause of inferior biofilm construction.

The production and components of the ECM of* A. fumigatus* have already been described in detail under both* in vitro* and* in vivo* conditions [5, 8]. ECM acts like a “glue,” packing the hyphae and enhancing biofilm formation by attaching to the host cells and substrates [23]. ECM can be observed using SEM when* A. fumigatus* is cultured in a medium with 10% fetal calf serum. SEM analysis showed that WT hyphae were surrounded with a membrane-like substance, which was not present in Δ*cspA* cultures. This substance has been predicted to be ECM [5]. Therefore, it is reasonable to suggest that* cspA* is associated with ECM production, and deletion of this gene severely affects biofilm formation.

As a growing number of patients are infected with pan-drug-resistant* A. fumigatus* strains, it is imperative to understand the resistance mechanisms and uncover novel drug targets [24]. Mowat et al. studied the effects of various antifungal drugs on several* A. fumigatus* strains and showed that biofilm formation produced a significant increase (>500-fold) in tolerance to these agents compared with planktonic cells [6]. In our study, 5-FC, which interferes with DNA synthesis, was the most effective antifungal agent for planktonic cells (PMIC 0.5 *μ*g/mL for WT). However, it had little effect once a biofilm was established, even at a 512-fold increase in concentration over the PMIC. On the other hand, AmB, which increases the permeability of the cell wall, inhibited biofilm development at a concentration only 2-fold higher than the PMIC. Therefore, a combination of 5-FC and AmB would be most effective for the treatment of* A. fumigatus* infection [25].

The Δ*cspA* strain showed increased susceptibility to 5-FC when in the planktonic state, while the sessile hyphae were more susceptible to AmB and ICZ compared with the WT strain. In WT cells, the ECM may prevent these molecules from penetrating into mycelia [26], meaning that the Δ*cspA* mutant, with its decreased production of ECM, may be more vulnerable to these antifungal agents.

Internalization by A549 lung alveolar epithelial cells is a model representing the invasiveness of fungi towards host cells and their ability to escape the immune system. Herein, we found that the Δ*cspA* mutant strain showed a 2-fold increase in internalization of conidia into lung alveolar epithelial cells compared with the WT strain. Little is known about the interaction between fungi and host nonimmunocytes. However, changes in the physical properties of conidia, such as hydrophobicity, surface roughness, and electrostatic charge, significantly affect cellular uptake [21]. Most cell lines, including A549, tend to absorb nanoparticles with a cationic charge and a rougher surface [27]. Previous studies have shown that carbohydrates with a negative charge can interfere with the adherence of conidia to connective components of pulmonary epithelial cells. The Δ*cspA* strain generated in the current study showed a lack of pigmentation (putatively a lack of melanin) and a rough surface and may have more chitin exposure (based on previous work [11]). Melanin confers a negative charge to conidia, while a chitin carries a cationic charge. These factors might indicate that the mutant strain carries a more positive charge than the WT [17, 28]. In addition, a* C. albicans* strain with increased exposure of chitin exhibited a stronger interaction with normal human gingival fibroblasts [29].

## 5. Conclusion

In this study, a Δ*cspA* mutant strain was achieved by targeted gene disruption mediated by* Agrobacterium tumefaciens*, and a reconstituted* cspA* strain was acquired by homologous introduction. Our results showed that deletion of* cspA* resulted in a lighter gray-green colony, rougher conidia surface, incomplete biofilm formation, and increased internalization into A549 cells, as well as enhanced sensitivity to some antifungal drugs, which suggests that* cspA* participates in maintaining the integrity of cell wall, plays an important role in biofilm formation, and may become a breakthrough point to understand the drug resistance mechanism of* A. fumigatus*.

## Figures and Tables

**Table 1:** Minimal inhibitory concentration (*µ*g/mL) of the *antifungal agents* against *A. fumigatus* strains after 24 hours of treatment *in vitro*.

Strains	Itraconazole			Amphotericin B			5-Fluorocytosine		
	PMIC	SMIC_50_	SMIC_90_	PMIC	SMIC_50_	SMIC_90_	PMIC	SMIC_50_	SMIC_90_
WT	0.5	8	16	16	32	64	0.5	>256	>256
Δ*cspA *	0.5	1	2	16	16	32	<0.125	>256	>256
*cspA*C	0.25	>32	>32	>16	32	64	0.5	>256	>256
